# Supplementary material for: Cell Penetrating Peptide Enhances the Aphidicidal Activity of Spider Venom-Derived Neurotoxin
Source: Toxins (Basel). 2024 Aug 14;16(8):358. doi: 10.3390/toxins16080358 (PMC11360749; doi:10.3390/toxins16080358)
Supplement: Supplementary file 1 [file toxins-16-00358-s001.zip › toxins-3140881-supp figures.pdf]

Supplementary Materials: Cell penetrating peptide enhances the aphidicidal activity of spider venom-derived neurotoxin

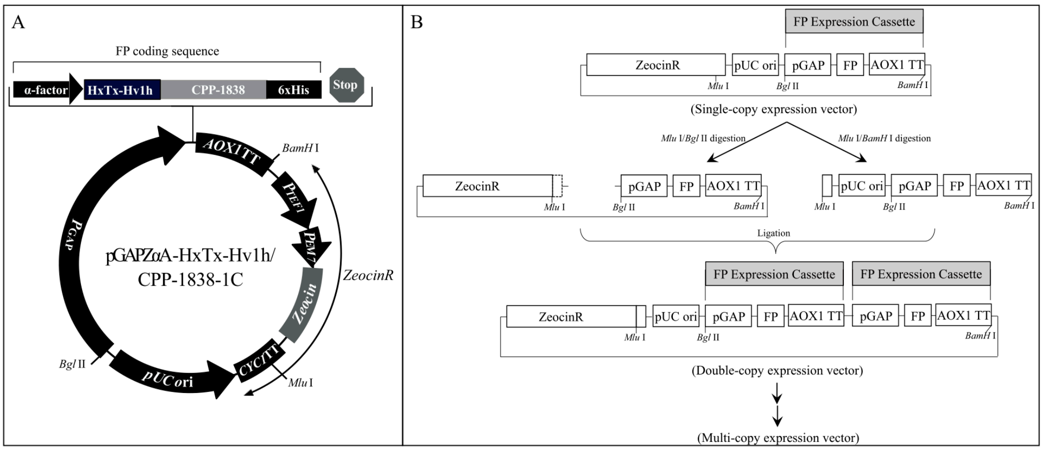

Supplementary Fig.S1. Diagrammatic overview of the strategy employed to construct vectors harboring multiple copies of the expression cassette. (A) Depiction of the specific insertion site for the HxTx-Hv1h/CPP-1838 cassette within the pGPZαA expression vector. (B) Schematic of the consecutive restriction-ligation steps which facilitate the serial integration of HxTx-Hv1h/CPP-1838 cassettes into the vector to generate a construct carrying multiple expression units. 'FP' refers to the HxTx-Hv1h/CPP-1838 fusion protein.

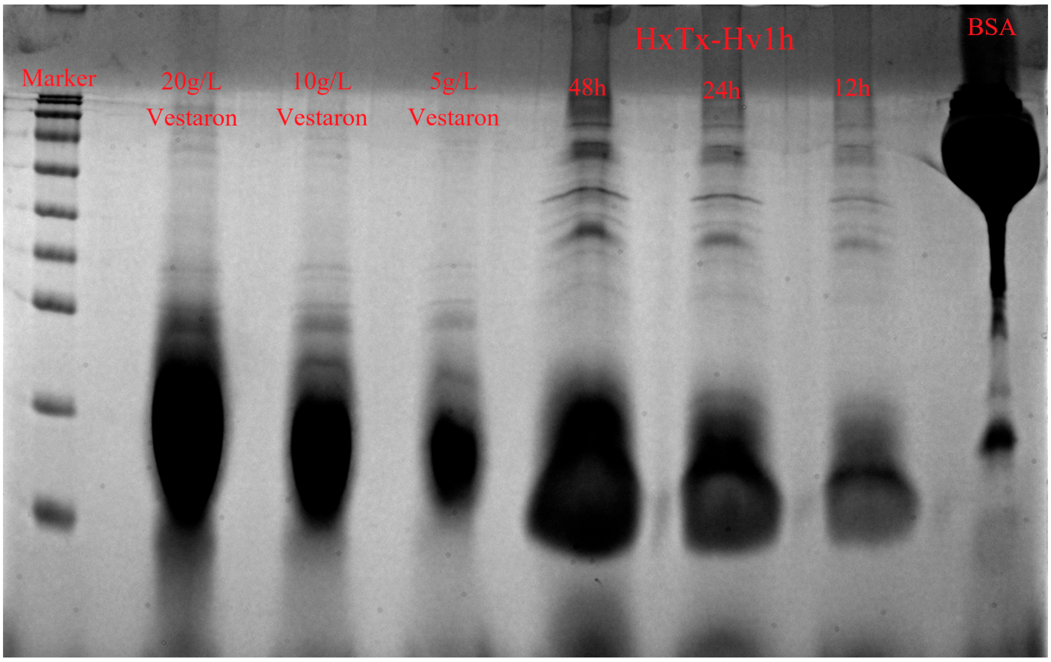

Supplementary Fig.S2. Quantitative assessment of HxTx-Hv1h recombinant protein production in *K. lactis* transformants. This figure illustrates the relative expression levels of the recombinant HxTx-Hv1h protein in *K. lactis* transformants. The commercially available HxTx-Hv1h from Vestaron's Spear® T product serves as a reference. Additionally, a 10 g/L bull serum albumin (BSA) standard is included for comparative analysis.
